# Squamous Cell Papilloma of the Oesophagus: A Human Papilloma Virus Lesion

**DOI:** 10.7759/cureus.19903

**Published:** 2021-11-25

**Authors:** Youssef Aladham, Omar Ahmed, Juliet Laycock

**Affiliations:** 1 Otolaryngology, East Kent Hospitals University NHS Foundation Trust, Ashford, GBR; 2 Otolaryngology, East Kent Hospitals University NHS foundation Trust, Ashford, GBR

**Keywords:** reflux, human papillomavirus (hpv), transnasal oesophagoscopy, oesophagus, squamous papilloma

## Abstract

Oesophageal squamous cell papilloma is a very rare entity with a limited number of reports in the literature. The exact aetiology is uncertain, and it commonly overlaps with gastro-oesophageal reflux. Human papilloma virus (HPV) is deemed responsible for some cases. Although incidental discovery during upper gastrointestinal endoscopy for other reasons is the commonest presentation, symptomatic cases do occur. Endoscopic excision is the standard treatment. We report a case of HPV-positive squamous papilloma of the upper oesophagus, presenting with lateralising throat pain and diagnosed with office transnasal oesophagoscopy. We also discuss features of HPV-positive oesophageal squamous papilloma and the role of transnasal oesophagoscopy as a recent diagnostic modality of increasing popularity.

## Introduction

Oesophageal squamous cell papilloma (OSP) is a rare benign epithelial lesion that is usually asymptomatic but can present with a spectrum of upper gastrointestinal symptoms [1]. With no gender predilection, the disease is commonly diagnosed in the forth and fifth decades of life [2]. The lesion is typically solitary, ranging from 2 to 6 mm in size, with around two-thirds of the cases localised to the lower third of the oesophagus. Symptomatic patients commonly have reflux-type symptoms with heartburn and epigastric discomfort mimicking gastro-oesophageal reflux (GORD), which commonly co-exists as such. Proximal lesions may present with throat symptoms, similar to those of laryngopharyngeal reflux. Endoscopic excision is the modality of treatment with complete remission in most cases [3].

## Case presentation

A 38-year-old female patient presented to our fast-track ENT clinic with persistent left-sided throat discomfort and pain for several months. The throat pain lateralised to the left and radiated to her left ear. She also reported positive reflux symptoms with heartburn. At the time of review, medical management had been implemented (with high-dose oral proton pump inhibitor and raft-forming alginate); however, this yielded no symptomatic relief. Voice Handicap Index (VHI) was 11 and Eating Assessment Tool (EAT-10) score was 1. She was a non-smoker, and her past medical history was insignificant.

**Figure 1:**
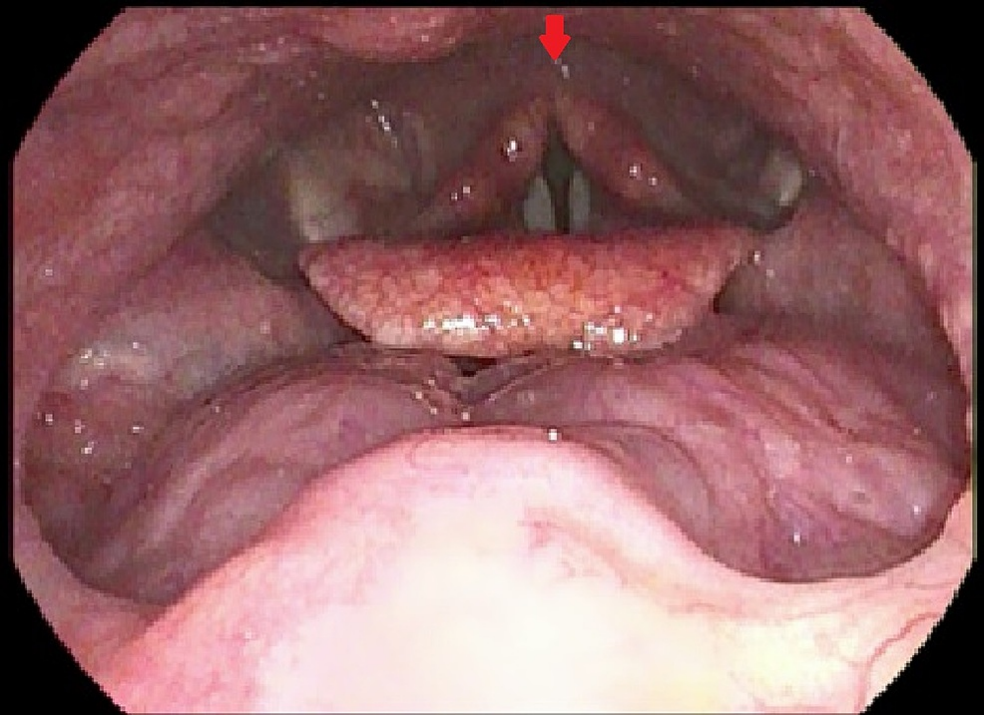
Flexible nasendoscopy showing no discrete lesions but relative post-cricoid oedema, a sign of laryngopharyngeal reflux. Red arrow indicates post-cricoid oedema.

**Figure 2:**
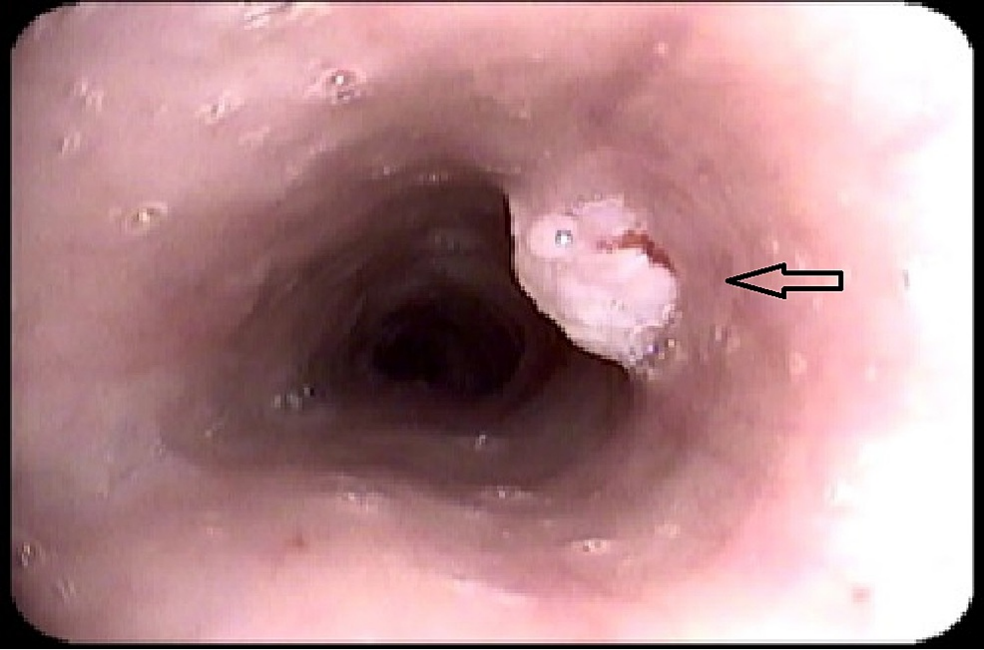
An endoscopic view of a polypoid sessile lesion with papillomatous surface on the left side of the proximal oesophagus. Picture was taken during transnasal oesophagoscopy. Arrow indicates the lesion.

Office examination of the neck, oral cavity and oropharynx was unremarkable. Flexible nasendoscopy was performed, which revealed some post-cricoid oedema consistent with laryngopharyngeal reflux (Figure 1). A contrast-enhanced magnetic resonance imaging (MRI) scan of the neck was normal. As symptoms continued to persist, a transnasal oesophagoscopy (TNO) under local anaesthetic was performed as an office procedure where a proximal oesophageal lesion was identified (Figure 2). The lesion appeared small (approximately 3 mm in diameter), exophytic, non-ulcerating and papillomatous. It was located on the left side of the upper oesophageal mucosa, 23 cm from the nasal sill. Multiple punch biopsies were taken.

Histological examination of the specimen revealed squamous cell papilloma of benign appearance with prominent papillae and acanthotic stratified squamous epithelium without cellular atypia. The specimen tested positive for human papilloma virus (HPV). Oesophageal manometry and 24-hour oesophageal pH monitoring indicated borderline pathological reflux with some proximal penetration (DeMeester score [DMS] = 15.9).

The patient then underwent complete endoscopic excision of the lesion. Histology confirmed HPV-positive squamous papilloma. Anti-reflux treatment, including lifestyle changes, was continued. Following excision, the patient’s symptoms resolved and a yearly follow-up confirmed their complete remission. It is yet uncertain whether symptomatic relief was a result of excision, continued anti-reflux treatment, or even a placebo effect associating the procedure.

## Discussion

The prevalence of OSP has been reported to range from 0.01% to 0.45% [1]. However, this figure is amongst symptomatic patients undergoing oesophagoscopy, and the actual population prevalence is unknown and likely to be much lower. That said, most of the diagnoses of OSP are made incidentally, and the lesions themselves rarely cause symptoms.

The exact aetiology of OSP is not yet certain with multiple proposed theories including chronic irritation as with GORD, mechanical and chemical injury, smoking and alcohol [4]. These factors result in longstanding mucosal irritation with hyper-regenerative response. This could account for why nearly two-thirds of OSP lesions are located in the distal third of the oesophagus, a site where maximal exposure to reflux-induced irritation occurs [2]. Additionally, HPV was isolated from OSP in several studies [5]. It is known that HPV infection is linked to some sexual behaviours, such as multiple sexual partners. Takeshita et al. studied 38 OSP in Japanese population, of which 10% tested positive for HPV [6]. Those HPV-positive papillomas were located in the mid-oesophagus and showed strong female predominance at a relatively young age. They posited that HPV-negative OSP (GORD-related) tends to involve the distal oesophagus, whilst HPV-positive OSP occurs more proximally. We note that our patient is a relatively young female with an HPV-positive proximal OSP, which supports that postulation.

Histologically, neutrophils have been identified in papilloma biopsies taken from the lower oesophagus; however, no neutrophils have been identified in papilloma biopsies from the mid or upper oesophagus [6]. In our patient, no neutrophilic infiltration of the stroma was noted. The theory of chronic inflammation is therefore less likely to be a major factor in the patient presented here. That said, the process may still be multifactorial and synergistic.

Whilst OSP is a benign lesion, its malignant potential is still debatable. Some cases of malignant transformation have been reported. In a cohort of 78 patients with OSP, 1.3% of lesions developed into squamous cell carcinoma at two-year follow-up endoscopy [7]. The margin required to remove the lesion remains controversial, as does the case for ongoing endoscopic surveillance. It is possible that HPV has a carcinogenic effect on OSP [8]. Further understanding of this relationship could further inform management decision of this rare entity.

The use of TNO in the office setting as a diagnostic tool for persistent upper aerodigestive symptoms has gained wide popularity recently. It has been shown to be a sensitive tool for small lesions, even with normal imaging [9]. It is noted that in our case, a neck MRI did not show the lesion when TNO clearly did. This highlights the importance of the use of TNO to investigate patients with unremitting upper aerodigestive symptoms.

## Conclusions

Patients with persistent unilateral throat pain and discomfort should be investigated for upper aerodigestive lesions. Laryngopharyngeal reflux commonly co-exists with different pharyngeal and oesophageal pathologies, which need to be excluded if there is no response to reflux treatment. TNO is an excellent diagnostic modality for direct visualisation of oesophageal mucosa, particularly in very small lesions that are commonly missed by MRI. Whilst malignant neoplasms of the oesophagus are much more common, benign oesophageal squamous papillomas have been reported.
